# AYUMS: an algorithm for completely automatic quantitation based on LC-MS/MS proteome data and its application to the analysis of signal transduction

**DOI:** 10.1186/1471-2105-8-15

**Published:** 2007-01-18

**Authors:** Ayumu Saito, Masao Nagasaki, Masaaki Oyama, Hiroko Kozuka-Hata, Kentaro Semba, Sumio Sugano, Tadashi Yamamoto, Satoru Miyano

**Affiliations:** 1 The Institute of Medical Science, The University of Tokyo, 4-6-1 Shirokanedai, Minato-ku, Tokyo 108-8639, Japan

## Abstract

**Background:**

Comprehensive description of the behavior of cellular components in a quantitative manner is essential for systematic understanding of biological events. Recent LC-MS/MS (tandem mass spectrometry coupled with liquid chromatography) technology, in combination with the SILAC (Stable Isotope Labeling by Amino acids in Cell culture) method, has enabled us to make relative quantitation at the proteome level. The recent report by Blagoev et al. (Nat. Biotechnol., **22**, 1139–1145, 2004) indicated that this method was also applicable for the time-course analysis of cellular signaling events. Relative quatitation can easily be performed by calculating the ratio of peak intensities corresponding to differentially labeled peptides in the MS spectrum. As currently available software requires some GUI applications and is time-consuming, it is not suitable for processing large-scale proteome data.

**Results:**

To resolve this difficulty, we developed an algorithm that automatically detects the peaks in each spectrum. Using this algorithm, we developed a software tool named AYUMS that automatically identifies the peaks corresponding to differentially labeled peptides, compares these peaks, calculates each of the peak ratios in mixed samples, and integrates them into one data sheet. This software has enabled us to dramatically save time for generation of the final report.

**Conclusion:**

AYUMS is a useful software tool for comprehensive quantitation of the proteome data generated by LC-MS/MS analysis. This software was developed using Java and runs on Linux, Windows, and Mac OS X. Please contact ayums@ims.u-tokyo.ac.jp if you are interested in the application. The project web page is .

## Background

The LC-MS/MS system is one of the most frequently used instruments for shotgun protein identification [1-6]. Protein identification by LC-MS/MS analysis consists mainly of the following five steps: (i) The samples are prepared from protein mixtures by peptide fragmentation with a protease, e.g., trypsin. (ii) In the LC column, the digested peptides are separated according to their hydrophobicity and/or polarity (iii) In the survey scan (MS-1) mode, the peptides eluted from the LC system are continuously introduced into the mass spectrometer by electrospray ionization (ESI). (iv) The detector in the MS-1 mode separates peptides according to the mass/charge ratio (m/z) and selects the peaks with high intensity. (v) In the MS/MS (MS-2) mode, the selected peptides are separated from other components and randomly fragmented by physical impact. The detector integrates the intensity of each fragment, leading to the generation of MS/MS spectra.

Recent development of quantitative proteomics technology has made it possible to perform quantitative analysis of large-scale proteome data generated using the LC-MS/MS system. SILAC (Stable Isotope Labeling by Amino acids in Cell culture) is one of the most effective methods for comparative analysis of the expression status of proteins among samples [7-10], including time-course analysis [11]. The SILAC method has undergone some modifications. One of the well-modified SILAC methods is as follows: (i) Target cells are incubated in three types of media, namely, media containing (1) natural arginine, (2) arginine containing stable isotope of ^13^C, or (3) arginine with two types of stable isotopes, ^13^C and ^15^N. (ii) The samples prepared from differentially labeled cells are mixed in equal proportions and introduced into the LC-MS/MS system. (iii) The peak derived from the same amino acid sequence is shifted in proportion to the difference of the number of neutrons between the samples. Relative quantitation can be performed by comparing the peak intensities of differentially labeled peptides [11].

The above method is widely used for describing various biological events [10-12]. For example, Blagoev et al. reported the global quantitative dynamics of phosphotyrosine-based signaling events by measuring the fold activation of related proteins at different time points [11].

Several types of software, e.g., SEQUEST [13], MOWSE [14], Mascot [15], ProteinProspector [16], and ProFound [17], have been developed for protein identification based on MS or MS/MS data. These software tools deduce a corresponding protein/peptide sequence from the measured data and generate a report with additional information, e.g. reliability score, gene ID, and modification if any. For quantitation, MZmine version 0.60 was developed for differential analyses of the LC/MS profile data [18]. Although this software uses a GUI interface with a powerful batch-processing function, its application is restricted to the analyses of LC/MS data. For further analyses using LC-MS/MS in combination with the SILAC method, MSQuant [19] has been developed. MSQuant has a GUI interface and runs on Windows OS. However, this software is not in stable operation and requires a huge memory (e.g., 2 GB) to run.

In the present study, we have developed a completely automatic console-based software tool that is highly customized for LC-MS/MS proteome data obtained by the SILAC method. Here we report a new algorithm for peak detection, details of the data analysis pipeline, and a new platform-independent open source software, AYUMS, developed using this algorithm. Furthermore, we compare the results obtained by manual operation with those obtained using this software and discuss the respective performances.

## Implementation

AYUMS consists of a series of steps for processing LC-MS/MS data. The scope of this software is focused on data processing for extracting quantitative information from the raw data. Therefore, other tools should be used for the statistical analyses based on the information produced by AYUMS. This software is implemented as a stand-alone Java application and requires JRE 1.4.2 or higher version. In contrast to MSQuant (which runs only on Windows), AYUMS is platform-independent, i.e., it runs on any of Windows, Unix, or Mac OS X. In addition, the generation of the final report is completely automatic.

### Software design

**Figure 1:**
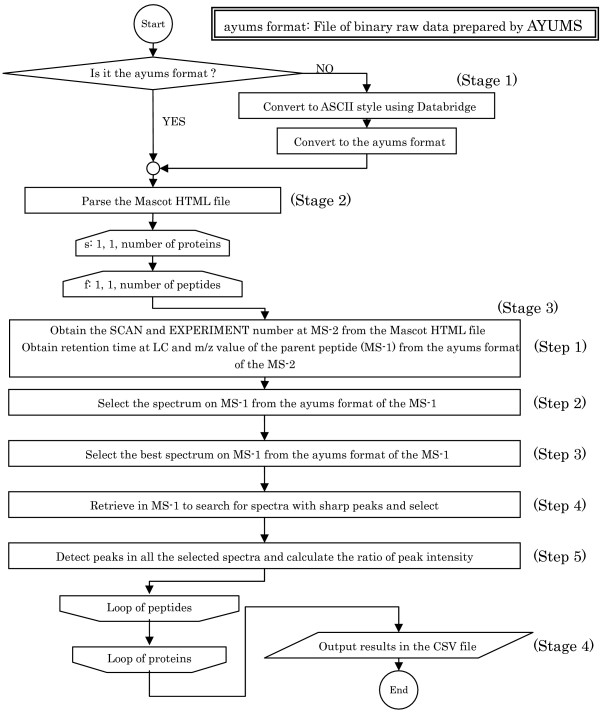
**Flow chart of AYUMS**. The procedure for AYUMS is illustrated in the flow chart. It consists of four stages: Stage 1, generation of an MS binary file, Stage 2, parsing of Mascot HTML, Stage 3, analysis of the spectrum data, Stage 4, generation of the analyzed reports. Stage 3 is subdivided into five steps, as described above.

Our aim was to develop a software tool that automatically executes the calculation of the peak ratios of differentially labeled peptides analyzed by LC-MS/MS. To achieve this, we adopted a console-based user interface (CUI). AYUMS requires two input files – an LC-MS/MS raw data file and a database search result file containing the information on the identified peptides/proteins. AYUMS generates an output report in a comma-separated value (CSV) format. The flow chart of AYUMS is shown in Figure 1 and the contents of the flow chart are described in the following sections.

### Input data style and conversion of the raw data file

**Figure 2:**
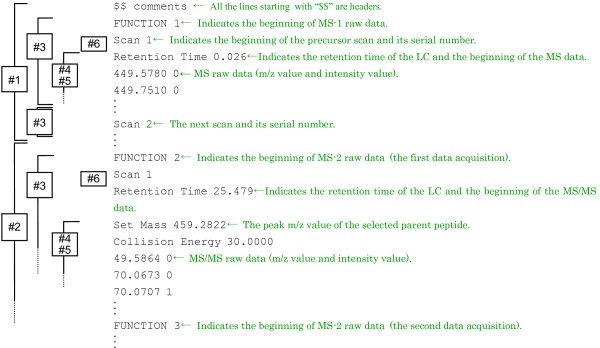
**Example of an ASCII format generated by Databridge**. Databridge generates an ASCII format from the raw data of LC-MS/MS analysis. The file comprises five blocks that start from the string FUNCTION 1–5. As shown above, the block that starts from FUNCTION 1 corresponds to the MS-1 raw data (#1), and the other four blocks correspond to the MS-2 raw data (#2). Each block has data on multiple spectra that start from the Scan (#3), which contains m/z values (#4), intensity values represented by integers (#5), and retention time of the LC (#6).

In the first stage (Stage 1 in Figure 1), AYUMS requires two files, namely, (i) a Mascot HTML file and (ii) a binary file in our original format (ayums format). For generating the Mascot HTML file, a peak list file is first prepared from the raw MS/MS data file using ProteinLynx (Micromass, UK). This peak list is searched against the protein database using Mascot (Matrix Science, UK) and the output of the database search is saved as an HTML file. The binary file is generated by the following two steps: first, the MassLynx raw data are converted to ASCII style data using Databridge in the MassLynx package (the format is shown in Figure 2); subsequently, this ASCII data file is converted to the ayums format using the conversion functions in AYUMS. Using a Pentium 4 (3.0 GHz) processor, the total time required for the conversion from the raw data to the ASCII style by Databridge is 30 min to 1 h, and the time from the ASCII style to the ayums format by AYUMS is 3 to 6 h.

### Parsing of Mascot HTML

The Mascot HTML file mainly comprises a list of inferred proteins and their peptides along with the information on the observed molecular weight, the calculated molecular weight, the difference between these two weights, probability-based Mowse score, p-value of the score, rank of the matched ion, peptide sequence, and MS/MS spectrum. In Stage 2, the Mascot HTML file is parsed to make these data available in AYUMS. The CyberNeko Java library developed by Andy Clark is used as an HTML parser [20]. If the XML format is implemented for the output of Mascot, an XML parser library will also be useful.

### Selection of reliable proteins and their peptides

In Stage 3, every matched protein and the list of identified peptides under the defined conditions are extracted from the parsed results of the Mascot HTML data. The criteria for data extraction are as follows: (i) select protein/peptides with a Mascot score higher than a threshold value, (ii) select peptides in higher ranks than a threshold value. The default condition in AYUMS is set to select all the peptides with a score higher than 25 in the top rank.

### Peak detection and computation

In Stage 4, the peaks corresponding to the selected peptides are searched from the raw data and the peak ratios of the differentially labeled peptides are calculated.

The following five steps are applied for each selected peptide.

#### Step 1

Based on the Mascot data of the selected peptide, the retention time at LC and the m/z value of the peptide are searched from the ayums format of MS-2.

#### Step 2

According to the information on the retention time obtained in Step 1, the nearest time point is searched from the ayums format of MS-1, leading to the acquisition of the spectrum corresponding to the target peptide.

#### Step 3

The spectra around this time point are sequentially searched. A specific algorithm, the details of which are described below, calculates a score for each spectrum and selects the best spectrum.

**Figure 3:**
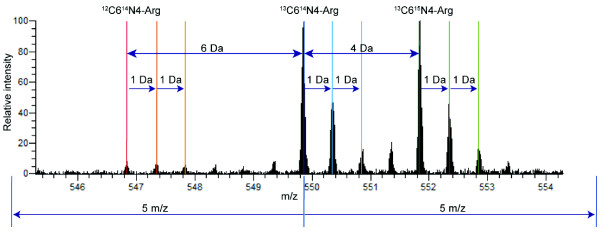
**Example of a spectrum at MS-1**. In the analysis using the SILAC method, three differential peak clusters are observed based on the mass difference of the stable isotopes introduced into the peptide sequence. In the above spectrum of a doubly charged peptide with an m/z value of 549.86, the highest ^13^C6^14^N4-Arg peak was analyzed for protein identification. Each peak cluster contains some additional peaks that derive from natural isotopes.

The spectrum consists of a set of peaks with each individual m/z value and intensity. All the intensities within a certain range of m/z value (default 0.1) from the target peak are integrated. Each peptide is differentially displayed in three distinct forms that are derived from three types of stably labeled arginine (^12^C^14^N, ^13^C^14^N, and ^13^C^15^N). According to the information in the Mascot result, the identified peptide form and its differentially labeled ones are specified in the spectrum based on the principle that the differences of molecular weight between ^12^C^14^N - ^13^C^14^N and ^13^C^14^N - ^13^C^15^N are 6Da and 4Da, respectively (Figure 3).

In addition, as proteins/peptides are made of some natural isotopes, each peak is accompanied by sub-peaks which shift 1 Da and 2 Da in the spectrum. The intensities of these peaks are all integrated as the total quantity of the target peptide.

#### Step 4

The spectra adjacent to the best spectrum are recursively selected as long as the score ratio of the investigated spectrum to the best one is higher than a constant value (default 0.8), which we term the acceptable ratio. Based on the data of the acceptable spectra, the intensities for three types of differentially labeled peptides are independently integrated.

#### Step 5

Based on the result in Step 4, the average ratios of ^13^C^14^N and ^13^C^15^N to ^12^C^14^N and their standard deviations are calculated.

### Algorithm

The procedure for Step 1 to Step 5 is described in the following algorithm.

*n *:= 1.008665

*r *:= 0.1

*r*_2 _:= 5.000

*r*_3 _:= 3

*r*_4 _:= 10

*r*_5 _:= -0.2

**for ***s *∈ *S*: set of protein

   **for **{(*f*_*i*_, *n*_*i*_, *c*_*i*_)|0≤*i*≤*N*} ∈ *F*(*s*): *F *is a function from a protein to the fragments of the protein, the scan number of the MS/MS experiment, and charge of each fragment.

      (*r*_*ms/ms*_, *mz*_*ms*_) := *R*_*ms/ms *_(*n*_*i*_) : *R*_*ms/ms *_is a function from a scan number of the MS/MS experiment to the MS/MS retention time and m/z value of MS experiment; these can be obtained from the raw data.

      (*r*_*ms*_, *n*_*ms*_) := *R*_*ms *_(*r*_*ms/ms*_): *R*_*ms *_is a function from an MS/MS retention time to the nearest MS retention time and its scan number.

      *e*_max _= 0, (Pt,j,max⁡1,Pt,j,max⁡2,Pt,j,max⁡3)
 MathType@MTEF@5@5@+=feaafiart1ev1aaatCvAUfKttLearuWrP9MDH5MBPbIqV92AaeXatLxBI9gBaebbnrfifHhDYfgasaacH8akY=wiFfYdH8Gipec8Eeeu0xXdbba9frFj0=OqFfea0dXdd9vqai=hGuQ8kuc9pgc9s8qqaq=dirpe0xb9q8qiLsFr0=vr0=vr0dc8meaabaqaciaacaGaaeqabaqabeGadaaakeaacqGGOaakcqWGqbaudaqhaaWcbaGaemiDaqNaeiilaWIaemOAaOMaeiilaWIagiyBa0MaeiyyaeMaeiiEaGhabaGaeGymaedaaOGaeiilaWIaemiuaa1aa0baaSqaaiabdsha0jabcYcaSiabdQgaQjabcYcaSiGbc2gaTjabcggaHjabcIha4bqaaiabikdaYaaakiabcYcaSiabdcfaqnaaDaaaleaacqWG0baDcqGGSaalcqWGQbGAcqGGSaalcyGGTbqBcqGGHbqycqGG4baEaeaacqaIZaWmaaGccqGGPaqkaaa@5130@, *m*_max _= 0, *t*_max _= ()

      **for **{*m*|*n*_*ms *_- *r*_3 _≤ *m *≤ *n*_*ms *_+ *r*_3_}

         (Pt,j,m1,Pt,j,m2,Pt,j,m3
 MathType@MTEF@5@5@+=feaafiart1ev1aaatCvAUfKttLearuWrP9MDH5MBPbIqV92AaeXatLxBI9gBaebbnrfifHhDYfgasaacH8akY=wiFfYdH8Gipec8Eeeu0xXdbba9frFj0=OqFfea0dXdd9vqai=hGuQ8kuc9pgc9s8qqaq=dirpe0xb9q8qiLsFr0=vr0=vr0dc8meaabaqaciaacaGaaeqabaqabeGadaaakeaacqWGqbaudaqhaaWcbaGaemiDaqNaeiilaWIaemOAaOMaeiilaWIaemyBa0gabaGaeGymaedaaOGaeiilaWIaemiuaa1aa0baaSqaaiabdsha0jabcYcaSiabdQgaQjabcYcaSiabd2gaTbqaaiabikdaYaaakiabcYcaSiabdcfaqnaaDaaaleaacqWG0baDcqGGSaalcqWGQbGAcqGGSaalcqWGTbqBaeaacqaIZaWmaaaaaa@472B@, *L*_*rate,m*_) := *sub*(*m*, *mz*_*ms*_, *c*_*i*_, *f*_*i*_): calculate the total intensities of a peak and its ratio in the spectrum.

         **if ***e*_max _<*L*_*rate,m*_

            *e*_max _:= *L*_*rate,m*_, *m*_max _:= *m*

            *t*_max _= (Pt,j,max⁡1,Pt,j,max⁡2,Pt,j,max⁡3)
 MathType@MTEF@5@5@+=feaafiart1ev1aaatCvAUfKttLearuWrP9MDH5MBPbIqV92AaeXatLxBI9gBaebbnrfifHhDYfgasaacH8akY=wiFfYdH8Gipec8Eeeu0xXdbba9frFj0=OqFfea0dXdd9vqai=hGuQ8kuc9pgc9s8qqaq=dirpe0xb9q8qiLsFr0=vr0=vr0dc8meaabaqaciaacaGaaeqabaqabeGadaaakeaacqGGOaakcqWGqbaudaqhaaWcbaGaemiDaqNaeiilaWIaemOAaOMaeiilaWIagiyBa0MaeiyyaeMaeiiEaGhabaGaeGymaedaaOGaeiilaWIaemiuaa1aa0baaSqaaiabdsha0jabcYcaSiabdQgaQjabcYcaSiGbc2gaTjabcggaHjabcIha4bqaaiabikdaYaaakiabcYcaSiabdcfaqnaaDaaaleaacqWG0baDcqGGSaalcqWGQbGAcqGGSaalcyGGTbqBcqGGHbqycqGG4baEaeaacqaIZaWmaaGccqGGPaqkaaa@5130@ := (pt,j,m1,pt,j,m2,pt,j,m3
 MathType@MTEF@5@5@+=feaafiart1ev1aaatCvAUfKttLearuWrP9MDH5MBPbIqV92AaeXatLxBI9gBaebbnrfifHhDYfgasaacH8akY=wiFfYdH8Gipec8Eeeu0xXdbba9frFj0=OqFfea0dXdd9vqai=hGuQ8kuc9pgc9s8qqaq=dirpe0xb9q8qiLsFr0=vr0=vr0dc8meaabaqaciaacaGaaeqabaqabeGadaaakeaacqWGWbaCdaqhaaWcbaGaemiDaqNaeiilaWIaemOAaOMaeiilaWIaemyBa0gabaGaeGymaedaaOGaeiilaWIaemiCaa3aa0baaSqaaiabdsha0jabcYcaSiabdQgaQjabcYcaSiabd2gaTbqaaiabikdaYaaakiabcYcaSiabdchaWnaaDaaaleaacqWG0baDcqGGSaalcqWGQbGAcqGGSaalcqWGTbqBaeaacqaIZaWmaaaaaa@47EB@)

         **end**

      **end**

      *T *= {*t*_max_}

      **for **{*m*|*m*_max _+ 1 ≤ *m *≤ *m*_max _+ *r*_4_}

         *t *= (pt,j,m1,pt,j,m2,pt,j,m3
 MathType@MTEF@5@5@+=feaafiart1ev1aaatCvAUfKttLearuWrP9MDH5MBPbIqV92AaeXatLxBI9gBaebbnrfifHhDYfgasaacH8akY=wiFfYdH8Gipec8Eeeu0xXdbba9frFj0=OqFfea0dXdd9vqai=hGuQ8kuc9pgc9s8qqaq=dirpe0xb9q8qiLsFr0=vr0=vr0dc8meaabaqaciaacaGaaeqabaqabeGadaaakeaacqWGWbaCdaqhaaWcbaGaemiDaqNaeiilaWIaemOAaOMaeiilaWIaemyBa0gabaGaeGymaedaaOGaeiilaWIaemiCaa3aa0baaSqaaiabdsha0jabcYcaSiabdQgaQjabcYcaSiabd2gaTbqaaiabikdaYaaakiabcYcaSiabdchaWnaaDaaaleaacqWG0baDcqGGSaalcqWGQbGAcqGGSaalcqWGTbqBaeaacqaIZaWmaaaaaa@47EB@, *L*_*rate,m*_) := *sub*(*m*, *mz*_*ms*_, *c*_*i*_, *f*_*i*_)

         **if ***e*_max _× (1 + *r*_5_) ≤ *L*_*rate,m*_

            **add ***t ***to ***T*

         **else**

            **break**

         **end**

      **end**

      **for **{*m*|1≤*m*≤*r*_4_}

         *t *= (pt,j,m1,pt,j,m2,pt,j,m3
 MathType@MTEF@5@5@+=feaafiart1ev1aaatCvAUfKttLearuWrP9MDH5MBPbIqV92AaeXatLxBI9gBaebbnrfifHhDYfgasaacH8akY=wiFfYdH8Gipec8Eeeu0xXdbba9frFj0=OqFfea0dXdd9vqai=hGuQ8kuc9pgc9s8qqaq=dirpe0xb9q8qiLsFr0=vr0=vr0dc8meaabaqaciaacaGaaeqabaqabeGadaaakeaacqWGWbaCdaqhaaWcbaGaemiDaqNaeiilaWIaemOAaOMaeiilaWIaemyBa0gabaGaeGymaedaaOGaeiilaWIaemiCaa3aa0baaSqaaiabdsha0jabcYcaSiabdQgaQjabcYcaSiabd2gaTbqaaiabikdaYaaakiabcYcaSiabdchaWnaaDaaaleaacqWG0baDcqGGSaalcqWGQbGAcqGGSaalcqWGTbqBaeaacqaIZaWmaaaaaa@47EB@, *L*_*rate,m*_) := *sub*(*m*_max _- *m*, *mz*_*ms*_, *c*_*i*_, *f*_*i*_)

         **if ***e*_max _× (1 + *r*_5_) ≤ *L*_*rate,m*_

            **add ***t ***to ***T*

         **else**

            **break**

         **end**

      **end**

(pratio,i1,pratio,i2):=(∑(pt,j1,pt,j2,pt,j3,L)∈Tpt,j2pt,j1/|T|,∑(pt,j1,pt,j2,pt,j3,L)∈Tpt,j3pt,j1/|T|)
 MathType@MTEF@5@5@+=feaafiart1ev1aaatCvAUfKttLearuWrP9MDH5MBPbIqV92AaeXatLxBI9gBaebbnrfifHhDYfgasaacH8akY=wiFfYdH8Gipec8Eeeu0xXdbba9frFj0=OqFfea0dXdd9vqai=hGuQ8kuc9pgc9s8qqaq=dirpe0xb9q8qiLsFr0=vr0=vr0dc8meaabaqaciaacaGaaeqabaqabeGadaaakeaacqGGOaakcqWGWbaCdaqhaaWcbaGaemOCaiNaemyyaeMaemiDaqNaemyAaKMaem4Ba8MaeiilaWIaemyAaKgabaGaeGymaedaaOGaeiilaWIaemiCaa3aa0baaSqaaiabdkhaYjabdggaHjabdsha0jabdMgaPjabd+gaVjabcYcaSiabdMgaPbqaaiabikdaYaaakiabcMcaPiabcQda6iabg2da9maabmaabaWaaSGbaeaadaaeqbqaamaalaaabaGaemiCaa3aa0baaSqaaiabdsha0jabcYcaSiabdQgaQbqaaiabikdaYaaaaOqaaiabdchaWnaaDaaaleaacqWG0baDcqGGSaalcqWGQbGAaeaacqaIXaqmaaaaaaqaaiabcIcaOiabdchaWnaaDaaameaacqWG0baDcqGGSaalcqWGQbGAaeaacqaIXaqmaaWccqGGSaalcqWGWbaCdaqhaaadbaGaemiDaqNaeiilaWIaemOAaOgabaGaeGOmaidaaSGaeiilaWIaemiCaa3aa0baaWqaaiabdsha0jabcYcaSiabdQgaQbqaaiabiodaZaaaliabcYcaSiabdYeamjabcMcaPiabgIGiolabdsfaubqab0GaeyyeIuoaaOqaamaaemaabaGaemivaqfacaGLhWUaayjcSdaaaiabcYcaSmaalyaabaWaaabuaeaadaWcaaqaaiabdchaWnaaDaaaleaacqWG0baDcqGGSaalcqWGQbGAaeaacqaIZaWmaaaakeaacqWGWbaCdaqhaaWcbaGaemiDaqNaeiilaWIaemOAaOgabaGaeGymaedaaaaaaeaacqGGOaakcqWGWbaCdaqhaaadbaGaemiDaqNaeiilaWIaemOAaOgabaGaeGymaedaaSGaeiilaWIaemiCaa3aa0baaWqaaiabdsha0jabcYcaSiabdQgaQbqaaiabikdaYaaaliabcYcaSiabdchaWnaaDaaameaacqWG0baDcqGGSaalcqWGQbGAaeaacqaIZaWmaaWccqGGSaalcqWGmbatcqGGPaqkcqGHiiIZcqWGubavaeqaniabggHiLdaakeaadaabdaqaaiabdsfaubGaay5bSlaawIa7aaaaaiaawIcacaGLPaaaaaa@A713@

end

      Qs1:=∑0≤i≤Npratio,i1N
 MathType@MTEF@5@5@+=feaafiart1ev1aaatCvAUfKttLearuWrP9MDH5MBPbIqV92AaeXatLxBI9gBaebbnrfifHhDYfgasaacH8akY=wiFfYdH8Gipec8Eeeu0xXdbba9frFj0=OqFfea0dXdd9vqai=hGuQ8kuc9pgc9s8qqaq=dirpe0xb9q8qiLsFr0=vr0=vr0dc8meaabaqaciaacaGaaeqabaqabeGadaaakeaacqWGrbqudaqhaaWcbaGaem4CamhabaGaeGymaedaaOGaeiOoaOJaeyypa0ZaaSGaaeaadaaeqbqaaiabdchaWnaaDaaaleaacqWGYbGCcqWGHbqycqWG0baDcqWGPbqAcqWGVbWBcqGGSaalcqWGPbqAaeaacqaIXaqmaaaabaGaeGimaaJaeyizImQaemyAaKMaeyizImQaemOta4eabeqdcqGHris5aaGcbaGaemOta4eaaaaa@484C@ : Qs1
 MathType@MTEF@5@5@+=feaafiart1ev1aaatCvAUfKttLearuWrP9MDH5MBPbIqV92AaeXatLxBI9gBaebbnrfifHhDYfgasaacH8akY=wiFfYdH8Gipec8Eeeu0xXdbba9frFj0=OqFfea0dXdd9vqai=hGuQ8kuc9pgc9s8qqaq=dirpe0xb9q8qiLsFr0=vr0=vr0dc8meaabaqaciaacaGaaeqabaqabeGadaaakeaacqWGrbqudaqhaaWcbaGaem4CamhabaGaeGymaedaaaaa@3063@ is the ratio of the amount of the wild type to the ^13^C^14^N form.

      Qs2:=∑0≤i≤Npratio,i2N
 MathType@MTEF@5@5@+=feaafiart1ev1aaatCvAUfKttLearuWrP9MDH5MBPbIqV92AaeXatLxBI9gBaebbnrfifHhDYfgasaacH8akY=wiFfYdH8Gipec8Eeeu0xXdbba9frFj0=OqFfea0dXdd9vqai=hGuQ8kuc9pgc9s8qqaq=dirpe0xb9q8qiLsFr0=vr0=vr0dc8meaabaqaciaacaGaaeqabaqabeGadaaakeaacqWGrbqudaqhaaWcbaGaem4CamhabaGaeGOmaidaaOGaeiOoaOJaeyypa0ZaaSGaaeaadaaeqbqaaiabdchaWnaaDaaaleaacqWGYbGCcqWGHbqycqWG0baDcqWGPbqAcqWGVbWBcqGGSaalcqWGPbqAaeaacqaIYaGmaaaabaGaeGimaaJaeyizImQaemyAaKMaeyizImQaemOta4eabeqdcqGHris5aaGcbaGaemOta4eaaaaa@4850@ : Qs2
 MathType@MTEF@5@5@+=feaafiart1ev1aaatCvAUfKttLearuWrP9MDH5MBPbIqV92AaeXatLxBI9gBaebbnrfifHhDYfgasaacH8akY=wiFfYdH8Gipec8Eeeu0xXdbba9frFj0=OqFfea0dXdd9vqai=hGuQ8kuc9pgc9s8qqaq=dirpe0xb9q8qiLsFr0=vr0=vr0dc8meaabaqaciaacaGaaeqabaqabeGadaaakeaacqWGrbqudaqhaaWcbaGaem4CamhabaGaeGOmaidaaaaa@3065@ is the ratio of the amount of the wild type to the ^13^C^15^N form.

      SDs1
 MathType@MTEF@5@5@+=feaafiart1ev1aaatCvAUfKttLearuWrP9MDH5MBPbIqV92AaeXatLxBI9gBaebbnrfifHhDYfgasaacH8akY=wiFfYdH8Gipec8Eeeu0xXdbba9frFj0=OqFfea0dXdd9vqai=hGuQ8kuc9pgc9s8qqaq=dirpe0xb9q8qiLsFr0=vr0=vr0dc8meaabaqaciaacaGaaeqabaqabeGadaaakeaacqWGtbWucqWGebardaqhaaWcbaGaem4CamhabaGaeGymaedaaaaa@3178@ := Standard deviation of {pratio,i1
 MathType@MTEF@5@5@+=feaafiart1ev1aaatCvAUfKttLearuWrP9MDH5MBPbIqV92AaeXatLxBI9gBaebbnrfifHhDYfgasaacH8akY=wiFfYdH8Gipec8Eeeu0xXdbba9frFj0=OqFfea0dXdd9vqai=hGuQ8kuc9pgc9s8qqaq=dirpe0xb9q8qiLsFr0=vr0=vr0dc8meaabaqaciaacaGaaeqabaqabeGadaaakeaacqWGWbaCdaqhaaWcbaGaemOCaiNaemyyaeMaemiDaqNaemyAaKMaem4Ba8MaeiilaWIaemyAaKgabaGaeGymaedaaaaa@3858@|0≤*i*≤*N*}

      SDs2
 MathType@MTEF@5@5@+=feaafiart1ev1aaatCvAUfKttLearuWrP9MDH5MBPbIqV92AaeXatLxBI9gBaebbnrfifHhDYfgasaacH8akY=wiFfYdH8Gipec8Eeeu0xXdbba9frFj0=OqFfea0dXdd9vqai=hGuQ8kuc9pgc9s8qqaq=dirpe0xb9q8qiLsFr0=vr0=vr0dc8meaabaqaciaacaGaaeqabaqabeGadaaakeaacqWGtbWucqWGebardaqhaaWcbaGaem4CamhabaGaeGOmaidaaaaa@317A@ := Standard deviation of {pratio,i2
 MathType@MTEF@5@5@+=feaafiart1ev1aaatCvAUfKttLearuWrP9MDH5MBPbIqV92AaeXatLxBI9gBaebbnrfifHhDYfgasaacH8akY=wiFfYdH8Gipec8Eeeu0xXdbba9frFj0=OqFfea0dXdd9vqai=hGuQ8kuc9pgc9s8qqaq=dirpe0xb9q8qiLsFr0=vr0=vr0dc8meaabaqaciaacaGaaeqabaqabeGadaaakeaacqWGWbaCdaqhaaWcbaGaemOCaiNaemyyaeMaemiDaqNaemyAaKMaem4Ba8MaeiilaWIaemyAaKgabaGaeGOmaidaaaaa@385A@|0≤*i*≤*N*}

end

**function ***sub*(*n*_*ms*_, *mz*_*ms*_, *c*, *f*)

      *L *= {(*t*_*m/z,j*_, *p*_*j*_)|0≤*j*≤*M*} := *P*(*n*_*ms *_): *P *is a function from an MS scan number to the set of m/z and its intensity values. This set can be searched from the raw data.

      *R *:= the number of arginine in *f*

      **if ***f *contains ^13^C and does not contain ^15^N

         mzms3
 MathType@MTEF@5@5@+=feaafiart1ev1aaatCvAUfKttLearuWrP9MDH5MBPbIqV92AaeXatLxBI9gBaebbnrfifHhDYfgasaacH8akY=wiFfYdH8Gipec8Eeeu0xXdbba9frFj0=OqFfea0dXdd9vqai=hGuQ8kuc9pgc9s8qqaq=dirpe0xb9q8qiLsFr0=vr0=vr0dc8meaabaqaciaacaGaaeqabaqabeGadaaakeaacqWGTbqBcqWG6bGEdaqhaaWcbaGaemyBa0Maem4CamhabaGaeG4mamdaaaaa@337F@ := *mz*_*ms *_+ 4*nR*/*c*

         mzms1
 MathType@MTEF@5@5@+=feaafiart1ev1aaatCvAUfKttLearuWrP9MDH5MBPbIqV92AaeXatLxBI9gBaebbnrfifHhDYfgasaacH8akY=wiFfYdH8Gipec8Eeeu0xXdbba9frFj0=OqFfea0dXdd9vqai=hGuQ8kuc9pgc9s8qqaq=dirpe0xb9q8qiLsFr0=vr0=vr0dc8meaabaqaciaacaGaaeqabaqabeGadaaakeaacqWGTbqBcqWG6bGEdaqhaaWcbaGaemyBa0Maem4CamhabaGaeGymaedaaaaa@337B@ := *mz*_*ms *_- 6*nR*/*c*

         mzms2
 MathType@MTEF@5@5@+=feaafiart1ev1aaatCvAUfKttLearuWrP9MDH5MBPbIqV92AaeXatLxBI9gBaebbnrfifHhDYfgasaacH8akY=wiFfYdH8Gipec8Eeeu0xXdbba9frFj0=OqFfea0dXdd9vqai=hGuQ8kuc9pgc9s8qqaq=dirpe0xb9q8qiLsFr0=vr0=vr0dc8meaabaqaciaacaGaaeqabaqabeGadaaakeaacqWGTbqBcqWG6bGEdaqhaaWcbaGaemyBa0Maem4CamhabaGaeGOmaidaaaaa@337D@ := *mz*_*ms*_

      **else if ***f *contains ^13^C and ^15^N

         mzms2
 MathType@MTEF@5@5@+=feaafiart1ev1aaatCvAUfKttLearuWrP9MDH5MBPbIqV92AaeXatLxBI9gBaebbnrfifHhDYfgasaacH8akY=wiFfYdH8Gipec8Eeeu0xXdbba9frFj0=OqFfea0dXdd9vqai=hGuQ8kuc9pgc9s8qqaq=dirpe0xb9q8qiLsFr0=vr0=vr0dc8meaabaqaciaacaGaaeqabaqabeGadaaakeaacqWGTbqBcqWG6bGEdaqhaaWcbaGaemyBa0Maem4CamhabaGaeGOmaidaaaaa@337D@ := *mz*_*ms *_- 4*nR*/*c*

         mzms1
 MathType@MTEF@5@5@+=feaafiart1ev1aaatCvAUfKttLearuWrP9MDH5MBPbIqV92AaeXatLxBI9gBaebbnrfifHhDYfgasaacH8akY=wiFfYdH8Gipec8Eeeu0xXdbba9frFj0=OqFfea0dXdd9vqai=hGuQ8kuc9pgc9s8qqaq=dirpe0xb9q8qiLsFr0=vr0=vr0dc8meaabaqaciaacaGaaeqabaqabeGadaaakeaacqWGTbqBcqWG6bGEdaqhaaWcbaGaemyBa0Maem4CamhabaGaeGymaedaaaaa@337B@ := *mz*_*ms *_- 10*nR*/*c*

         mzms3
 MathType@MTEF@5@5@+=feaafiart1ev1aaatCvAUfKttLearuWrP9MDH5MBPbIqV92AaeXatLxBI9gBaebbnrfifHhDYfgasaacH8akY=wiFfYdH8Gipec8Eeeu0xXdbba9frFj0=OqFfea0dXdd9vqai=hGuQ8kuc9pgc9s8qqaq=dirpe0xb9q8qiLsFr0=vr0=vr0dc8meaabaqaciaacaGaaeqabaqabeGadaaakeaacqWGTbqBcqWG6bGEdaqhaaWcbaGaemyBa0Maem4CamhabaGaeG4mamdaaaaa@337F@ := *mz*_*ms*_

      **end**

      Pt,j1
 MathType@MTEF@5@5@+=feaafiart1ev1aaatCvAUfKttLearuWrP9MDH5MBPbIqV92AaeXatLxBI9gBaebbnrfifHhDYfgasaacH8akY=wiFfYdH8Gipec8Eeeu0xXdbba9frFj0=OqFfea0dXdd9vqai=hGuQ8kuc9pgc9s8qqaq=dirpe0xb9q8qiLsFr0=vr0=vr0dc8meaabaqaciaacaGaaeqabaqabeGadaaakeaacqWGqbaudaqhaaWcbaGaemiDaqNaeiilaWIaemOAaOgabaGaeGymaedaaaaa@32A0@ := *peakIntensitySet*(mzms1
 MathType@MTEF@5@5@+=feaafiart1ev1aaatCvAUfKttLearuWrP9MDH5MBPbIqV92AaeXatLxBI9gBaebbnrfifHhDYfgasaacH8akY=wiFfYdH8Gipec8Eeeu0xXdbba9frFj0=OqFfea0dXdd9vqai=hGuQ8kuc9pgc9s8qqaq=dirpe0xb9q8qiLsFr0=vr0=vr0dc8meaabaqaciaacaGaaeqabaqabeGadaaakeaacqWGTbqBcqWG6bGEdaqhaaWcbaGaemyBa0Maem4CamhabaGaeGymaedaaaaa@337B@, *L*, *r*, *c*)

      Pt,j2
 MathType@MTEF@5@5@+=feaafiart1ev1aaatCvAUfKttLearuWrP9MDH5MBPbIqV92AaeXatLxBI9gBaebbnrfifHhDYfgasaacH8akY=wiFfYdH8Gipec8Eeeu0xXdbba9frFj0=OqFfea0dXdd9vqai=hGuQ8kuc9pgc9s8qqaq=dirpe0xb9q8qiLsFr0=vr0=vr0dc8meaabaqaciaacaGaaeqabaqabeGadaaakeaacqWGqbaudaqhaaWcbaGaemiDaqNaeiilaWIaemOAaOgabaGaeGOmaidaaaaa@32A2@ := *peakIntensitySet*(mzms2
 MathType@MTEF@5@5@+=feaafiart1ev1aaatCvAUfKttLearuWrP9MDH5MBPbIqV92AaeXatLxBI9gBaebbnrfifHhDYfgasaacH8akY=wiFfYdH8Gipec8Eeeu0xXdbba9frFj0=OqFfea0dXdd9vqai=hGuQ8kuc9pgc9s8qqaq=dirpe0xb9q8qiLsFr0=vr0=vr0dc8meaabaqaciaacaGaaeqabaqabeGadaaakeaacqWGTbqBcqWG6bGEdaqhaaWcbaGaemyBa0Maem4CamhabaGaeGOmaidaaaaa@337D@, *L*, *r*, *c*)

      Pt,j3
 MathType@MTEF@5@5@+=feaafiart1ev1aaatCvAUfKttLearuWrP9MDH5MBPbIqV92AaeXatLxBI9gBaebbnrfifHhDYfgasaacH8akY=wiFfYdH8Gipec8Eeeu0xXdbba9frFj0=OqFfea0dXdd9vqai=hGuQ8kuc9pgc9s8qqaq=dirpe0xb9q8qiLsFr0=vr0=vr0dc8meaabaqaciaacaGaaeqabaqabeGadaaakeaacqWGqbaudaqhaaWcbaGaemiDaqNaeiilaWIaemOAaOgabaGaeG4mamdaaaaa@32A4@ := *peakIntensitySet*(mzms3
 MathType@MTEF@5@5@+=feaafiart1ev1aaatCvAUfKttLearuWrP9MDH5MBPbIqV92AaeXatLxBI9gBaebbnrfifHhDYfgasaacH8akY=wiFfYdH8Gipec8Eeeu0xXdbba9frFj0=OqFfea0dXdd9vqai=hGuQ8kuc9pgc9s8qqaq=dirpe0xb9q8qiLsFr0=vr0=vr0dc8meaabaqaciaacaGaaeqabaqabeGadaaakeaacqWGTbqBcqWG6bGEdaqhaaWcbaGaemyBa0Maem4CamhabaGaeG4mamdaaaaa@337F@, *L*, *r*, *c*)

      *L*_*total *_:= select all (*t*, *p*) ∈ *L *with [mzms2
 MathType@MTEF@5@5@+=feaafiart1ev1aaatCvAUfKttLearuWrP9MDH5MBPbIqV92AaeXatLxBI9gBaebbnrfifHhDYfgasaacH8akY=wiFfYdH8Gipec8Eeeu0xXdbba9frFj0=OqFfea0dXdd9vqai=hGuQ8kuc9pgc9s8qqaq=dirpe0xb9q8qiLsFr0=vr0=vr0dc8meaabaqaciaacaGaaeqabaqabeGadaaakeaacqWGTbqBcqWG6bGEdaqhaaWcbaGaemyBa0Maem4CamhabaGaeGOmaidaaaaa@337D@ - *r*_2 _≤ *t *≤ mzms2
 MathType@MTEF@5@5@+=feaafiart1ev1aaatCvAUfKttLearuWrP9MDH5MBPbIqV92AaeXatLxBI9gBaebbnrfifHhDYfgasaacH8akY=wiFfYdH8Gipec8Eeeu0xXdbba9frFj0=OqFfea0dXdd9vqai=hGuQ8kuc9pgc9s8qqaq=dirpe0xb9q8qiLsFr0=vr0=vr0dc8meaabaqaciaacaGaaeqabaqabeGadaaakeaacqWGTbqBcqWG6bGEdaqhaaWcbaGaemyBa0Maem4CamhabaGaeGOmaidaaaaa@337D@ + *r*_2_]

      **return **(Pt,j1,Pt,j2,Pt,j3,∑l=13Pt,jl/Ltotal
 MathType@MTEF@5@5@+=feaafiart1ev1aaatCvAUfKttLearuWrP9MDH5MBPbIqV92AaeXatLxBI9gBaebbnrfifHhDYfgasaacH8akY=wiFfYdH8Gipec8Eeeu0xXdbba9frFj0=OqFfea0dXdd9vqai=hGuQ8kuc9pgc9s8qqaq=dirpe0xb9q8qiLsFr0=vr0=vr0dc8meaabaqaciaacaGaaeqabaqabeGadaaakeaadaWcgaqaaiabdcfaqnaaDaaaleaacqWG0baDcqGGSaalcqWGQbGAaeaacqaIXaqmaaGccqGGSaalcqWGqbaudaqhaaWcbaGaemiDaqNaeiilaWIaemOAaOgabaGaeGOmaidaaOGaeiilaWIaemiuaa1aa0baaSqaaiabdsha0jabcYcaSiabdQgaQbqaaiabiodaZaaakiabcYcaSmaaqahabaGaemiuaa1aa0baaSqaaiabdsha0jabcYcaSiabdQgaQbqaaiabdYgaSbaaaeaacqWGSbaBcqGH9aqpcqaIXaqmaeaacqaIZaWma0GaeyyeIuoaaOqaaiabdYeamnaaBaaaleaacqWG0baDcqWGVbWBcqWG0baDcqWGHbqycqWGSbaBaeqaaaaaaaa@5695@)

end

**function ***peakIntensitySet*(*m*_*z*_, *L*, *r*, *c*)

      *L*':= select all (*t*, *p*) ∈ *L *with [*m*_*z *_- *r *≤ *t *≤ *m*_*z *_+ *r*]

      *L*'':= select all (*t*, *p*) ∈ *L *with [*m*_*z *_- *r *+ *n*/*c *≤ *t *≤ *m*_*z *_+ *r *+ *n*/*c*]

      *L*''':= select all (*t*, *p*) ∈ *L *with [*m*_*z *_- *r *+ 2*n*/*c *≤ *t *≤ *m*_*z *_+ *r *+ 2*n*/*c*]

      **return **∑(t,p)∈L′t+∑(t,p)∈L″t+∑(t,p)∈L‴t
 MathType@MTEF@5@5@+=feaafiart1ev1aaatCvAUfKttLearuWrP9MDH5MBPbIqV92AaeXatLxBI9gBaebbnrfifHhDYfgasaacH8akY=wiFfYdH8Gipec8Eeeu0xXdbba9frFj0=OqFfea0dXdd9vqai=hGuQ8kuc9pgc9s8qqaq=dirpe0xb9q8qiLsFr0=vr0=vr0dc8meaabaqaciaacaGaaeqabaqabeGadaaakeaadaaeqbqaaiabdsha0bWcbaGaeiikaGIaemiDaqNaeiilaWIaemiCaaNaeiykaKIaeyicI4SafmitaWKbauaaaeqaniabggHiLdGccqGHRaWkdaaeqbqaaiabdsha0bWcbaGaeiikaGIaemiDaqNaeiilaWIaemiCaaNaeiykaKIaeyicI4SafmitaWKbayaaaeqaniabggHiLdGccqGHRaWkdaaeqbqaaiabdsha0bWcbaGaeiikaGIaemiDaqNaeiilaWIaemiCaaNaeiykaKIaeyicI4SafmitaWKbaibaaeqaniabggHiLdaaaa@51A5@

end

### Results output

**Figure 4:**
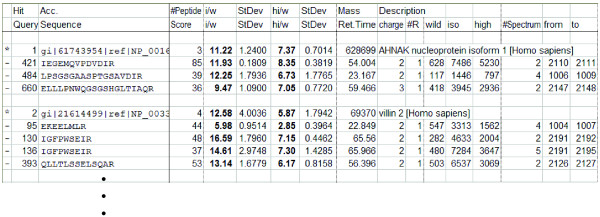
**The report style of AYUMS in the CVS format**. The final result is generated on a spreadsheet. The rows that start from "*" indicate the information on each protein (designated as Protein row). The subsequent rows that start from "-" show the data on each peptide (designated as Peptide row). The first two rows of the spreadsheet are the headers for Protein row and Peptide row, respectively. The columns of "i/w" and the next "StDev" are common to both the Protein and Peptide row, indicating the intensity ratio of ^13^C^14^N to ^12^C^14^N and its standard deviation, respectively. Similarly, those of "hi/w" and the next "StDev" indicate the ratio of ^13^C^15^N to ^12^C^14^N and its standard deviation, respectively. The "#Peptide" cell in the Protein row indicates the number of peptides for quantitative analysis in AYUMS. The "Mass" and "Description" cells indicate the molecular weight and the gene definition of the protein, respectively; these two contents are also described in the Mascot result file. The "Score" cell in the Peptide row indicates the Mascot score for each peptide. The "Ret.Time," "charge," and "#R" cells indicate the retention time, the charge, and the number of arginine residues for each peptide, respectively. The "wild," "iso," and "high" cells indicate the integrated peak intensities derived from ^12^C^14^N, ^13^C^14^N, and ^13^C^15^N, respectively. The "#Spectrum," "from," and "to" cells indicate the number of integrated spectra, the beginning and the end of the scan numbers used for data acquisition, respectively.

In Stage 4, AYUMS generates a report in the CSV file format, as shown in Figure 4. The contents of the report are also described in the legend for Figure 4.

## Results

### Comparison of the machine operation with the manual operation

**Figure 5:**
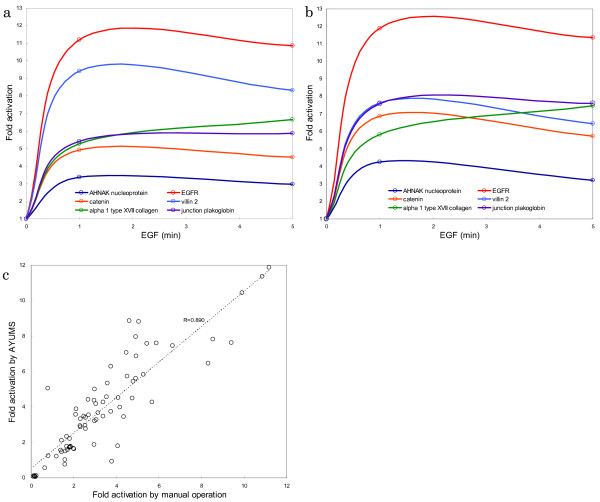
**Comparison of the results obtained using AYUMS versus manual operation**. The output performance of AYUMS and manual operation is compared based on the time-series proteome data of A431 cells stimulated with EGF. The observation points are 0 min, 1 min, and 5 min. The proteins with the top six Mascot scores were selected for the comparison. (a) 2D-Plot data of the output obtained by manual operation (x-axis: time, y-axis: fold activation of each protein). (b) 2D-Plot data of the output obtained by AYUMS. (c) The correlation chart of 38 phosphorylated proteins for 1-min and 5-min observation points between AYUMS and manual operation. The correlation coefficient was 0.890.

In order to evaluate the performance of the automatic calculation by AYUMS, we used three sets of time-series data on the phosphotyrosine-related proteome. A431 Cells differentially labeled with stable isotopes of arginine were stimulated with epidermal growth factor (EGF) for different time periods, followed by affinity-purification of signaling molecules with anti-phosphotyrosine antibodies. After direct digestion of the proteins, protein identification and quantification were performed by nanoLC-MS/MS analysis (nanoLC: Dina-2A [KYA Technologies]; tandem mass spectrometer: Q-Tof-2 [Micromass]). Figure 5 shows the activation profile of phosphorylated proteins with the top six Mascot scores (AHNAK nucleoprotein, EGFR, catenin, villin 2, alpha 1 type XVII collagen, and junction plakoglobin). Figures 5(a) and 5(b) show the results obtained by manual operation and by AYUMS, respectively. From the experimental data, 100 proteins were detected by database search against the RefSeq human protein database (NCBI). In the pre-process, our algorithm removed 62 proteins with a Mascot score less than the threshold (default; 25). The remaining 38 phosphorylated proteins were then quantified by manual operation as well as by AYUMS. As shown in Figure 5(c), the results obtained by these two methods showed good correlation (R = 0.890).

Although the results for some proteins did not correlate well (for example, the value for villin 2 obtained by AYUMS is lower than that obtained by manual operation), the shapes of the activation change between the two methods matched each other in most cases. It should be noted that AYUMS enabled us to eliminate the necessity for manual operation. In other words, reliable quantitation results were obtained in a high-throughput fashion that had never been achieved previously.

The poor correlation for some proteins was mainly due to the existence of noise peaks. The background noise has a substantial influence on quantitation, especially in the case of low-abundance peaks. The contaminant noise derived from other peptides also affects the calculation. Although our instrument operates with high mass resolution (10,000 FWHM) and accuracy (50~100 ppm), it is difficult to distinguish the other peaks with adjacent m/z values. Although it is possible to remove unreliable data when performing analysis manually, our algorithm does not have a function to eliminate them efficiently. Some statistical methods are necessary to deal with this problem.

## Discussion

### Reduction of difficulties

The major contributions of this study are as follows: (i) drastic reduction in the manual work required to perform quantitation for large-scale proteome data and (ii) reproducibility of high-quality data that does not depend on the user. In the case of this study, it required 2–6 working days to create the activation profile of the phosphotyrosine-related proteome by manual operation. In contrast, AYUMS could automatically generate the final report within 6 hours using a single machine. It is also possible to perform quantitation in parallel for multiple experimental data. For example, if two machines are available, 3 hours are sufficient for the generation of the final result.

Once the ayums format file is created, the subsequent analysis can be completed within 15 minutes. Thus, it is possible to easily re-evaluate experimental data by changing various options such as the acceptable ratio in Step 4 of Stage 3 and the threshold of the Mascot score.

### Future studies

Although a completely automatic quantitation based on the LC-MS/MS data was realized using AYUMS 1.0, further development of this software is required at various points. First, although the input of Stage 1 in AYUMS supports only the Q-tof type raw data, it needs to handle major data formats by NetCDF for more general purposes. Second, it would be very helpful to generate the final result not only in the CSV file format but also in other major formats, such as mzXML [21], for better usability.

The present SILAC method enables us to compare only two or three samples in a single experiment. Relative quantitation of target proteins at multiple points such as in dynamics analysis requires a common standard point to normalize the results of separate experiments. AYUMS will need to support a function of statistical data processing of the normalized results for more precise quantitation.

Although AYUMS is customized for the SILAC method, it can also easily handle the data obtained by other labeling strategies such as isotope-coded affinity tags (ICAT) [22], isobaric tags for relative and absolute quantitation (iTRAQ) [23], and culture-derived isotope tags (CDIT) [24].

This software is open to public access; hence, any researcher can contribute to the development of its application.

## Conclusion

AYUMS is a useful software tool for quantitative proteomics by LC-MS/MS technology in combination with the SILAC method. This software completely eliminates the need for manual work that has always been required previously. Besides, it enables us to obtain the final result considerably faster than by manual operation. Our evaluation of the output data by AYUMS indicated that it ranked comparably with the results calculated by an expert in proteomics.

## Availability and requirements

• Project home page: 

• Operating system(s): Java platform independent

• Programming language: Java

• Other requirements: Java 1.4.2 or higher, CyberNeko HTML Parser 0.9.5 or higher

• License: AYUMS software is available from the authors at ayums@ims.u-tokyo.ac.jp.

• Any restrictions to use by non-academics: Need contract.

## Authors' contributions

AS developed the new algorithms for peak recognition, operated the software, and wrote the manuscript. MN developed the new algorithms for peak recognition, helped to implement the algorithms, operate the software and prepare the manuscript. MO initiated this study, provided knowledge about the structure of the input raw data and wrote the manuscript. HK-H performed the experiment and helped to operate the software. KS provided knowledge about biochemistry. SS provided knowledge about proteomics technology. TY provided knowledge about signal transduction. SM supervised the dry study. All the authors read and approved of the final manuscript.
